# *Anisakis* Infection in the Spotted Flounder *Citharus linguatula* (Pleuronectiformes: Citharidae) Caught in the Gulf of Cadiz (Area FAO 27-ICES IXa) Appears to Negatively Affect Fish Growth

**DOI:** 10.3390/pathogens11121432

**Published:** 2022-11-28

**Authors:** Manuel Morales-Yuste, Waldo Sánchez-Yebra, Mario Garrido, Rocío Benítez, Francisco Javier Adroher

**Affiliations:** Departamento de Parasitología, Facultad de Farmacia, Universidad de Granada, 18071 Granada, Spain

**Keywords:** *Anisakis*, *Hysterothylacium*, spotted flounder, *Citharus linguatula*, Gulf of Cadiz, Spain, FAO 27.IXa, anisakiasis, fish growth

## Abstract

Spotted flounder (*Citharus linguatula* L.) caught in the Gulf of Cadiz (area FAO 27 ICES IXa) were examined for *Anisakis* larvae and to assess the possible risk of anisakiasis in humans through consumption of this fish. Larvae of the genera *Anisakis* and *Hysterothylacium* were identified in the analysis of 128 purchased fish specimens. All *Anisakis* larvae corresponded to type I. Molecular analysis showed the presence of *A. pegreffii*, *A. simplex* s.s., and recombinant genotype between the two. The prevalence of *Anisakis* was 9.4% with a mean intensity of 1.42, while for *Hysterothylacium* the values were 12.5% and 1.06. The length and weight of the fish, but not Fulton’s condition factor, varied significantly between infected and uninfected fish. The prevalence of *Anisakis* increased with fish length, with no fish parasitized with *Anisakis* measuring less than 15.5 cm (2–2.5 years old), which is probably related to the reported dietary change of these fish at around 2 years of age. Fish not parasitized with any of these nematodes showed positive allometric growth, while those parasitized only with *Anisakis* showed negative allometric growth. When comparing both groups including only fish ≥ 15.5 cm (the smallest size of *Anisakis*-infected fish), the difference is shown to be statistically significant (*p* = 0.01), suggesting that *Anisakis* infection of spotted flounder negatively affects fish growth even when parasite intensity is low, which may have important economic repercussions. Finally, the low prevalence and, above all, intensity of *Anisakis* in these fish, as well as the habit of consuming this fish fried in oil in our geographical area, means that the risk of acquiring anisakiasis through consumption of this fish is low.

## 1. Introduction

Anisakidosis or anisakiasis is infection by anisakid larvae, acquired through consumption of fish or cephalopods infected with viable third-stage larvae (L3). Anisakidae are a family of parasitic nematodes with a complex life cycle that includes some species which can accidentally infect humans, mostly of the genus *Anisakis* and, less commonly, of the genus *Pseudoterranova* [1]. These genera involve crustaceans, cephalopods, and marine fish as intermediate/paratenic hosts and marine mammals as definitive hosts. Rarely, the genera *Contracaecum* (*C. osculatum*) and *Hysterothylacium* (*H. aduncum*) (this now family Raphidascarididae) have been implicated in human infection [1]. 

*Citharus linguatula* (Linnaeus, 1758) (spotted flounder) is a marine flatfish of the order Pleuronectiformes and family Citharidae found along the entire eastern Atlantic coast to 23° S, as well as throughout the Mediterranean as far as the Black Sea. It is a demersal fish, with adults inhabiting soft bottoms from the shoreline to about 300 m depth, although rarely caught at depths greater than 200 m [2]. They feed on small fish and crustaceans [3]. Although it does not have the commercial value of other flatfish of the same size, such as the common sole (*Solea solea*), its lower price (approximately one-third of that of the common sole in our geographical area) makes it attractive to the consumer. In Andalusia (southern Spain), more than half a million kg of spotted flounder were marketed in the period of 2020 to 2021 [4].

It is a fish that has been little studied as a host of anisakids, but its significant consumption makes it necessary to clarify whether it can pose a risk to human health. For this purpose, the fish available in the fish markets of a city in southern Spain were taken as a reference. Moreover, from a biological and commercial point of view, it would be useful to know if this fish is affected by the presence of the parasite. For this purpose, Fulton’s condition factor and the exponent *b* of the potential equation relating fish length and weight were calculated. The study shows the modification of growth type in fish parasitized with *Anisakis* even when parasite intensity is low.

## 2. Materials and Methods

### 2.1. Host and Parasites

A total of 128 spotted flounder (*C. linguatula*) caught in the area of the Gulf of Cadiz (FAO 27, ICES IXa) between February and April 2022 were purchased in different fish markets in the city of Granada (southern Spain). They were immediately transported on ice to the laboratory where total length and total weight were individually recorded. They were then dissected, separating viscera and musculature, and processed independently for observation and collection of macroscopic nematodes. No other macroscopic parasites were observed on analysis of the visceral package and musculature. Microscopic parasites were not considered. Once this process was completed, and in order to detect hidden and/or intramuscular nematodes, the viscera were placed in labelled transparent plastic bags, and the musculature was filleted to a thickness of no more than 2 mm (approximately) and also placed in labelled bags. All bags were frozen at −20 °C for at least 24 h before being exposed to UV light for detection of dead anisakids [5]. Although Pippy [6], as early as 1970, observed differences in autofluorescence of frozen larvae of *Anisakis simplex* and *Hysterothylacium aduncum*, for this detection process, internal controls of *Anisakis* and *Hysterothylacium* were randomly included. For this purpose, an UV transilluminator at 366 nm was used. The morphological identification of the isolated nematodes was based on the characteristics previously described [7,8,9,10,11,12].

In order to determine the general health status of the fish, Fulton’s condition factor was calculated according to the following equation:

CF = 100 × W/L^3^, (1)

where W = weight (g) and L = length (cm), expressing the nutritional status of the fish [13]. It is also accepted that the relationship between length and weight of a fish is a potential equation of the type:

W = a·L*^b^* (2)

with the parameters a, coefficient, and *b*, exponent, to be determined. When the exponent *b* is close to 3, it means that the growth is isometric. If *b* is clearly different from 3, the growth of the fish is assumed to be allometric, positive if greater than 3 and negative if less [14].

### 2.2. Molecular Identification of Anisakis larvae

The *Anisakis* larvae found were individually subjected to polymerase chain reaction followed by restriction fragment length polymorphism (PCR-RFLP) as previously described [15,16,17], in order to perform specific identification using *A. simplex* s.s. and *A. pegreffii* as controls, since only *Anisakis* type I larvae were observed (*sensu* Berland [8]). The larval DNA was extracted using the Real-Pure kit, following the manufacturer’s instructions. This DNA was then amplified by PCR using NC5 (forward) and NC2 (reverse) primers [18], which yielded the expected result of an amplicon of about 1000 bp, corresponding to the ITS1-5.8S-ITS2 fragment of the rDNA. Next, an RFLP of the isolated DNA was performed, subjecting it to digestion with restriction enzymes (0.5 U/µl) *Hinf I* and *Taq I* at 37 and 65 °C, respectively, for 10 min, and subsequent 3% agarose gel electrophoresis to separate and reveal the enzyme-generated fragments, using the aforementioned controls to allow their specific identification [19,20]. In this regard, *A. simplex* s.s. generated 3 bands of 620, 250, and 100 bp for *Hinf I* and 3 bands of 430, 400, and 100 bp for *Taq I*, while *A. pegreffii* generated 3 bands of 370, 300, and 250 bp for *Hinf I* and 3 bands of 400, 320, and 150 bp for *Taq I*. When a banding pattern that was the sum of these two species for one or both enzymes was generated, they were considered as recombinant genotype larvae between the above two species. 

### 2.3. Epidemiological Parameters and Statistical Analysis

Epidemiological parameters of prevalence (P), mean intensity (MI), and mean abundance (MA), as described by Bush et al. [21], were determined using the free Quantitative Parasitology 3.0 software developed by Reiczigel & Rózsa [22], based on their theoretical work [23,24], which takes into account the notorious leftward bias of parasite distributions in their hosts. Using the same software, Fisher’s exact test was used to compare prevalence, while mean intensity and abundance were compared by a bootstrap 2-sample *t*-test (with 2,000 repetitions). To compare length, weight, and Fulton’s condition factor between fish groups, Student’s *t*-test was used. To identify possible outliers, the box plot was used and then confirmed with Grubb’s test. To assess whether infection status (parasitized vs. non-parasitized) determines the relationship between fish weight and fish length, we performed ANCOVA analyses based on natural logarithms, with ln weight as the response variable and infection status (factor), ln length (covariate), and its interaction as explanatory variables. To avoid spurious results derived from body length differences between groups, a complementary ANCOVA was performed considering only individuals whose length was equal to or higher than the smallest *Anisakis*-infected individual (15.5 cm). All statistical analyses, except those previously indicated, were conducted in R [25] using the car [26] and outliers [27] packages. Differences were considered significant when *p* ≤ 0.05.

## 3. Results

### 3.1. Parasitized and Non-Parasitized Fish

The 128 spotted flounder (*C. linguatula*) analysed had the following values ± standard deviation and (range): mean length was 15.25 ± 1.77 (10.9–19.3) cm, mean weight was 28.36 ± 10.32 (8.97–53.87) g, and mean Fulton’s condition factor (CF) (CF = 100·W/L^3^; where W is weight in g and L is length in cm) was 0.761 ± 0.076 (0.483–1.417) (Table 1). The relationship between fish weight and length was shown to be potential according to the equation y = a·x*^b^* (a = 0.0037 ± 0.0007; *b* = 3.2594 ± 0.0728; R^2^ = 0.9413). This exponent greater than 3 indicates a positive allometric growth of the sampled set of fish. When performing the box plot with CF to determine possible outliers, the fish with the lowest CF value (0.483; W = 22.11 g and L = 16.6 cm) was identified as an outlier. This was checked with the Grubb’s test, identified as a statistical outlier (*p* = 0.01) and removed to study fish growth.

**Figure 1:**
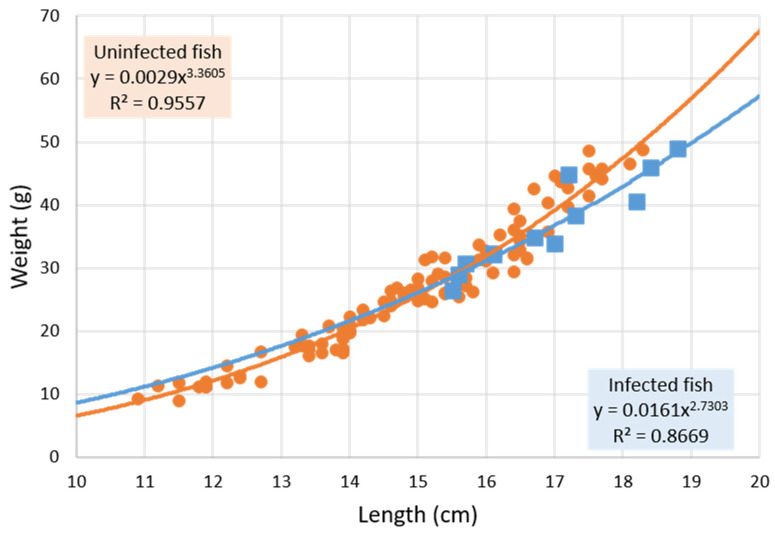
Potential weight–length relationship (y = a·x*^b^*) in non-*Anisakis*-infected fish (n = 101; *circle*) and *Anisakis*-infected *Citharus linguatula* (n = 12; *square*). Fish parasitized only with *Hysterothylacium* have been excluded. Uninfected ± SD: exponent *b* ± 0.0731, coefficient a ± 0.0006. Infected ± SD: exponent *b* ± 0.3340, coefficient a ± 0.0152.

**Figure 2:**
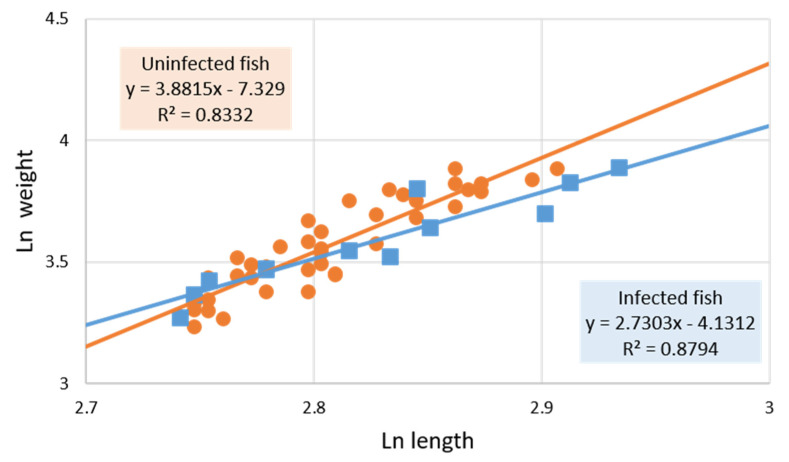
Logarithmic transformation of the relationship between weight and length of *Anisakis*-infected (n = 12; *square*) and non-*Anisakis*-infected *Citharus linguatula* (n = 41; *circle*), considering only individuals ≥ 15.5 cm (the length of the smallest *Anisakis*-infected fish). Fish parasitized only with *Hysterothylacium* have been excluded. ANCOVA statistical comparison shows that the curves are significantly different (*p* = 0.01). The value of *b* corresponds to the slope of the line.

However, when the exponent *b* was calculated separating *Anisakis*-parasitized and non-parasitized fish, it was observed (Figure 1 and Figure 2) that the former presented negative allometric growth (*b* < 3, *b* = 2.73) while the latter was positive (*b* > 3, *b* = 3.36), showing the modification the fish undergoes in growth type when parasitized by *Anisakis* larvae. ANCOVA results indicate that the relationship between fish weight and fish length differs between *Anisakis*-parasitized and non-parasitized individuals but is not significant, despite showing a trend. However, significance was obtained when considering only those individuals measuring at least 15.5 cm (the length of the smallest *Anisakis*-infected individual fish; ANCOVA, fish length * infection status: F_1, 49_ = 6.81; *p* = 0.01). Figure 2 shows that the set of fish ≥ 15.5 cm in length had higher positive allometric growth (*b* = 3.88) than the total set of fish not infected with *Anisakis* (*b* = 3.36; Figure 1). The same type of calculations performed for *Hysterothylacium* infection were not significant.

**Figure 3:**
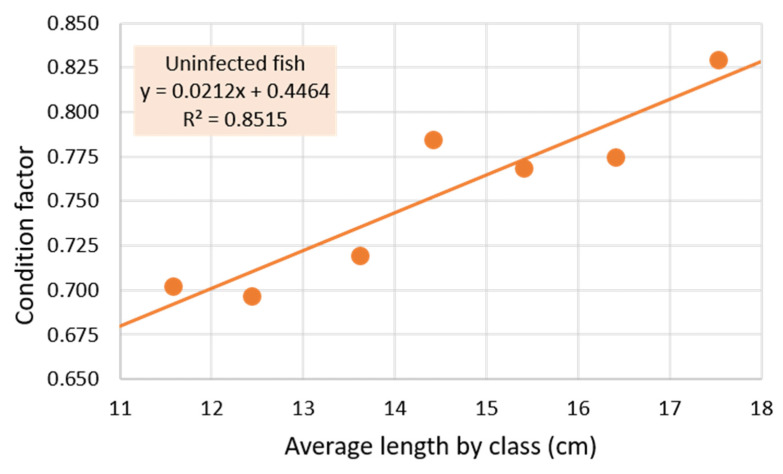
Relationship between mean length of uninfected *Citharus linguatula* length classes (<12 cm with 8 fish, 12–12.9 with 6, 13–13.9 with 16, 14–14.9 with 16, 15–15.9 with 24, 16–16.9 with 19, and ≥17 cm with 12 fish) and Fulton’s condition factor.

The mean condition factor of the uninfected set of fish was 0.762 ± 0.072, although it can be seen in Figure 3 that there is a trend of increasing CF with length (slope = +0.021), especially evident when comparing fish in the lower length classes (less than 14 cm), with CFs less than the mean, with those in the length classes (greater than 14 cm) that have CFs greater than the mean. When the CFs of the fish of these two groups are compared, a very high statistical significance is obtained (*p* < 1·10^−6^).

### 3.2. Parasites

Examination of the fish resulted in the detection of 34 nematode larvae, all of the superfamily Ascaridoidea. Of these, 17 were morphologically identified as L3 of *Anisakis* type I and the rest as larvae of the genus *Hysterothylacium*. All larvae were isolated from the viscera except one L3 *Anisakis* isolated from the musculature in one fish which, in addition, contained two other *Anisakis* larvae in the viscera, being the only host with an intensity higher than two. Thus, the prevalence in muscle was 0.8%.

Epidemiological parameters are shown in Table 1, with a prevalence of 9.4% for *Anisakis* and 12.5% for *Hysterothylacium*. Only two hosts were co-infected by *Anisakis* and *Hysterothylacium*, with one larva of each genus, all isolated in viscera.

**Figure 4:**
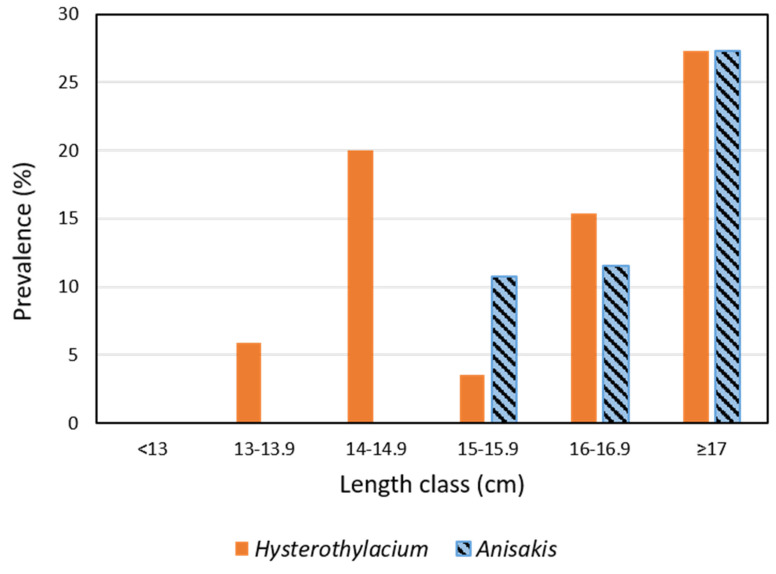
Prevalence of *Anisakis* and *Hysterothylacium* in *Citharus linguatula* by length class. The number of fish per length class, from left to right, are: 14, 17, 20, 28, 26, and 22.

In view of the results in Table 2, which show significant differences in length and weight of infected and uninfected fish but not in the condition factor, and Figure 4, which shows the highest prevalence of *Anisakis* in the longest fish (P = 27%, MI = 1.83, MA = 0.50), we calculated these parameters in these fish ≥17 cm and found that there were no significant differences in length and weight but that there were significant differences in the condition factor (*p* < 0.02). No significant differences in these parameters were found when comparing fish of smaller sizes. 

Since fish weight and length are two highly correlated variables (Pearson’s correlation 0.939), *Anisakis* infection was related to length only (Table 3). For this purpose, two groups of fish with a similar number of data were separated according to whether their length was less than 15.5 cm or ≥ 15.5 cm, which corresponds approximately to 2–2.5 years of life and to the sexual maturity of the fish [28,29]. The data in Table 3 show that larger fish are more parasitized by ascaridoids and *Anisakis* (both *p* < 0.001), but not significantly so in the case of *Hysterothylacium*.

### 3.3. Molecular Identification of Anisakis larvae

Of the 17 *Anisakis* type I larvae isolated, 16 were processed for genetic identification, obtaining eleven larvae of *A. pegreffii*, three of *A. simplex* s.s., and two of recombinant genotype between the two previous species (one only for *Taq I* and another for both enzymes). Only one larva was detected in muscle and identified as *A. pegreffii*.

## 4. Discussion

Parasitic nematodes of spotted flounder (*C. linguatula*) have been little studied, and only a few papers record ascaridoids, such as those belonging to the genera *Anisakis* (*A. simplex* s.s. and *A. pegreffii*), *Hysterothylacium* (*H. aduncum* and *H. fabri*), and some larvae of *Contracaecum* sp., mainly in waters of the Iberian Peninsula [30,31,32,33,34], but also in the Adriatic Sea [35,36]. 

Both *Hysterothylacium* and *Anisakis* larvae have been detected in this work. The prevalence is similar in both cases (Table 1), but only two fish were found to be parasitized by larvae of both genera, so the prevalence by ascaridoids is almost the sum of the two taxa detected (Table 1). The same is true for the mean abundance.

The spotted flounder is a marine flatfish inhabiting soft bottom habitats up to 300 m deep. Throughout its life, it feeds mainly on crustaceans, cephalopods, and fish from its area of influence, which may vary throughout its development. Among the crustaceans, caridean decapods and mysids are the most commonly consumed [37,38], although Belghyti et al. [39] reported a reduction in the consumption of polychaetes, cephalopods, and amphipods, with age associated with an increase in consumption of decapods and molluscs. However, they also reported many other complementary prey, among which copepods, the main first intermediate hosts of *Hysterothylacium* together with some hyperid amphipods [40], are not frequent. It can thus be suggested that *C. linguatula* is probably infected by this nematode via paratenic hosts that are predators of copepods; for example, crustaceans such as mysids, decapods, and amphipods, in addition to small fish [11]. This wide variety of spotted flounder prey, which can be hosts for *Hysterothylacium*, could explain the absence of significant differences associated with the size/age of the fish (Table 3), although there is a trend towards an increase in the epidemiological parameters, probably related to a cumulative effect of parasites in the fish with the consumption of infected prey (Table 3, Figure 4). On the other hand, euphausiids, the main first intermediate hosts of *Anisakis*, are considered complementary prey which are consumed mainly from 2 years of age onwards [39]. This could explain the significantly higher prevalence and mean abundance of *Anisakis* in longer/older fish (Table 3, Figure 4).

On the other hand, although no significant variations in the condition factor associated with parasitism were detected (Table 2), a modification in the growth of fish infected by *Anisakis* larvae was observed. This is a change from positive allometric growth in the uninfected fish to negative allometric growth in infected fish (Figure 1 and Figure 2). This trend becomes significant (*p* = 0.01) when comparing the growth of infected and uninfected fish ≥15.5 cm (size of the smallest fish infected with *Anisakis*; approximate age 2–2.5 years [28,29]).

Although the decrease in fish condition factor associated with high parasite intensity [41,42] that could lead to growth impairment is known [43], to our knowledge this is the first time that significant impairment of fish growth by *Anisakis* infection at such a low intensity (MI = 1.4, Table 1), without significant modification of the CF, has been reported. However, a significant reduction in CF was observed only in fish ≥17 cm infected with *Anisakis* (*p* < 0.02), which were those with the highest prevalence (Figure 4) and mean intensity (MI = 1.83). The condition factor is known to be affected by numerous factors, including the age and/or maturity of the fish (see Figure 3), the season, and the energy availability of the fish [44,45,46,47]. This is of vital economic importance for the fishing industry since, if confirmed in other fish of commercial interest, production yields, whether in commercial fisheries or in fish farming, can be reduced by even mild infections with *Anisakis*. This has already been shown in fish farms with higher intensities of this nematode (MI = 6.56 [48]). Although unknown factors cannot be ruled out in these changes in the growth of *Anisakis*-parasitized fish, the fact that they are from the same area and caught within a short period of time leads to the assumption that all fish have been subjected to the same or similar conditions, which makes a cause–effect relationship quite plausible, i.e., that the growth of the fish has been affected by *Anisakis* infection.

However, contrary to what might be expected from previous data, we have observed that parasitized fish have a significantly greater length and weight than uninfected fish (Table 2), as we have previously detected in other fish species parasitized with *Anisakis* [15,16]. This could be explained by the cumulative nature of these infections since, once the weakest fish are eliminated by predation and/or death, the strongest (and/or those with a lower parasite load) are selected and will attain a greater age, presumably presenting a higher resistance to infection due to having a more robust/mature immune system that allows the parasite and the host to coexist [1,44,45,49,50,51,52,53,54].

Finally, the molecular identification of *Anisakis* larvae showed the majority presence of *A. pegreffii* (68.75%) together with *A. simplex* s.s. (18.75%) and larvae with recombinant genotype between the two (12.50%). Although most of the coasts of the Iberian Peninsula are sympatric areas for these *Anisakis* species, the majority presence of *A. pegreffii* is common in the Gulf of Cadiz, decreasing in proportion as we move northward along the Atlantic coast of the Iberian Peninsula [55,56].

## 5. Conclusions

In summary, as both *A. pegreffii* and *A. simplex s.s.* are considered pathogenic species [57,58] and in view of the data reported here, there is a risk of *Anisakis* infection through consumption of raw or undercooked spotted flounder. However, this risk can be considered low due to the low prevalence and intensity of parasites in the fish, especially in the muscle, and the Andalusian culinary tradition of frying fish in oil.

Fish parasites affect the lives of their hosts by regulating their growth, reducing their fertility and affecting swimming, feeding, and behaviour [44,59] and thus their ability to survive in the ecosystem. The results of the present study are especially interesting, since they show a change in the type of growth of *Anisakis*-parasitized fish versus non-parasitized fish, even in the context of low parasitic intensity. This may have important implications for decision making within the fishing industry, as it may have a high economic impact, especially in the field of mariculture.

## Figures and Tables

**Table 1:** Epidemiological parameters for infection of *Citharus linguatula* by ascaridoids.

	Ascaridoidea	*Anisakis*	*Hysterothylacium*
Prevalence (%) (CI 95%)	20.3 (14.0–28.1)	9.4 (5.4–15.9)	12.5 (7.7–19.4)
Mean Intensity (range) (CI 95%)	1.31 (1–3) (1.12–1.50)	1.42 (1–3) (1.08–1.75)	1.06 (1–2) (1.00–1.19)
Mean Abundance (CI 95%)	0.27 (0.17–0.37)	0.13 (0.06–0.23)	0.13 (0.08–0.20)

Prevalence = 100·N/F; Mean intensity = A/N; Mean abundance = A/F, where F is the total number of fish, N is the number of infected fish, and A is the number of larvae. CI: confidence interval.

**Table 2:** Host parameters in *Citharus linguatula* according to nematode infection.

Host Parameters	Uninfected Fish	Fish Infected with		
		Ascaridoidea	*Anisakis*	*Hysterothylacium*
Number of fish	101	26	12	16
Mean length ± SD (cm)	14.93 ± 1.72	16.47 ± 1.45 **	16.88 ± 1.14 **	16.24 ± 1.55 *
Mean weight ± SD (g)	26.71 ± 10.01	35.02 ± 9.29 **	36.50 ± 7.19 *	34.46 ± 10.49 *
Condition factor ± SD	0.762 ± 0.072	0.768 ± 0.071 ^ns^	0.752 ± 0.054 ^ns^	0.783 ± 0.085 ^ns^

SD: standard deviation. Student’s *t*-test comparison of length, weight, and condition factor between uninfected and infected fish: * *p* ≤ 0.005; ** *p* < 0.0005; ^ns^, not significant.

**Table 3:** Epidemiological parameters of Ascaridoidea infection in spotted flounder *Citharus linguatula* by length classes.

Parasites	Parameters	<15.5 cm	≥15.5 cm
	Fish number	65	62
Ascaridoidea	Prevalence (%) (CI 95%)	7.7 (2.5–17.1)	33.9 ** (22.3–47.0)
	Mean Intensity (range) (CI 95%)	1.00 (1) uncertain	1.38 * (1–3) (1.14–1.62)
	Mean Abundance (CI 95%)	0.08 (0.02–0.14)	0.47 ** (0.29–0.65)
*Anisakis*	Prevalence (%) (CI 95%)	0 (0.00–5.52)	19.4 ** (10.4–31.4)
	Mean Intensity (range) (CI 95%)	0 n.a.	1.42 ^ns^ (1–3) (1.08–1.75)
	Mean Abundance (CI 95%)	0 uncertain	0.27 * (0.15–0.45)
*Hysterothylacium*	Prevalence (%) (CI 95%)	7.7 (2.5–17.1)	17.7 ^ns^ (9.2–29.5)
	Mean Intensity (range) (CI 95%)	1.00 (1) uncertain	1.09 ^ns^ (1–2) (1.00–1.27)
	Mean Abundance (CI 95%)	0.08 (0.02–0.14)	0.19 ^ns^ (0.10–0.31)

Prevalence = 100·N/F; Mean intensity = A/N; Mean abundance = A/F, where F is the total number of fish, N is the number of infected fish, and A is the number of larvae. CI: confidence interval; n.a.: not applicable. Student’s *t*-test comparison of prevalence and mean abundance by length classes showed significance for *Anisakis* and Ascaridoidea (* *p* < 0.05; ** *p* < 0.001). ^ns^ not significant.

## Data Availability

Not applicable. The datasets generated during and/or analyzed during the current study are all included in this manuscript.
